# Understanding significant experiences of adolescent athletes’ participation in competitive sports life: a systematic review

**DOI:** 10.3389/fspor.2025.1515200

**Published:** 2025-03-24

**Authors:** M. Fadli Dongoran, Heny Setyawati, Agus Kristiyanto, Hermawan Pamot Raharjo, Caly Setiawan

**Affiliations:** ^1^ Department of Physical Education, Faculty of Sports Science, Universitas Negeri Semarang, Semarang, Indonesia; ^2^ Doctoral Program in Sport Education (Postgraduated), Universitas Negeri Semarang, Semarang, Indonesia; ^3^ Department of Physical Education, Faculty of Sports, Universitas Sebelas Maret, Surakarta, Indonesia; ^4^ Department of Physical Education, Faculty of Sports Science, Universitas Negeri Yogyakarta, Yogyakarta, Indonesia

**Keywords:** youth athlete, athlete development, competitive life, youth experience, sports complexity

## Abstract

This research aims to investigate the significant experiences of adolescent athletes in competitive sports activities and their development through the available literature. This study systematically reviews research on adolescent athletes participating in competitive sports over the past 10 years. We evaluated and reported 19 studies in four sections: sample characteristics, research design, significant experiences, and key findings related to the development of adolescent athletes. This study includes adolescent athletes who participate in various sports and pursue careers in organized and competitive sports using qualitative and mixed methods designs. This study employs a systematic literature review as its research method. The initial online search was conducted in April 2023 and updated on January 2025 on electronic databases: Web of Science (WoS), PubMed, and Scopus. The process of searching for articles used the PRISMA guidelines proposed by Moher. We synthesized and interpreted the findings in each article. The main findings of this study lead to three main themes: meaningful experiences for adolescents, barriers in the development of adolescent athletes, and that influencing athlete development. This study concludes that working with adolescent athletes requires attention to unseen factors to ensure that they are in a supportive environment that encourages their positive development into elite athletes in the future.

## 1 Introduction

There is much dispute and complexity in understanding competitive sports in adolescents. Literature (1) explains that, on the surface, adolescents appear healthy and happy, with families reporting higher satisfaction levels when adolescents participate. On the other hand, competitive sports with pressure to win can damage the psychological and mental health of adolescents, not to mention the constant threat of injury and the high costs associated with participation in competitions. Competitive and organized sports, in many cases, aim to be a step towards Olympic elite sports or professional sports through increased competitiveness and professionalization (2). This trend confirms the increasing professionalization and specialization in youth sports, aiming to maximize the identification and development of talent for advancement to elite sports, a striking trend with potential physical and psychological consequences (3). The widely adopted trend of professionalization in youth sports training has generated various speculations (4–6). A survey estimates that 63.1% of Australian youth participate in organized sports (7). Thus, organized sports programs hold a legitimate place in Australian culture and have a significant role in youth development (8). In addition, millions of children around the world participate in community, school, and private sports programs (9).

Organized sports play an important role in the development of children and adolescents today. It has been proposed that sports can be an important mechanism for facilitating growth and well-being in young people (10). This is primarily because sports involve elements generally proven to positively affect mental health, such as physical activity and social engagement. In a systematic review in 2013, Eime and colleagues found many psychological and social health benefits from sports participation for young people, including increased self-esteem, social interaction, and reduced symptoms of depression (11). The physical and psychosocial benefits of involvement in sports are well recognized (12). This is reinforced by meta-analysis findings that show adolescents involved in sports report fewer symptoms of depression and anxiety-based symptoms compared to those who do not participate in sports. However, the effects are minor (13).

Unfortunately, adolescent athletes face competitive demands that can increase vulnerability to symptoms and mental health disorders during a developmental period or puberty, which is already challenging with emotional, cognitive, and behavioral changes (14, 15). Based on the mental health survey, also known as the Young Minds Matter Survey, conducted in Australia in (2013–14), it was reported that 14% of all children and adolescents (aged 4–17 years) experienced mental health disorders in the previous 12 months (8, 15). Common mental health disorders among adolescents include attention deficit hyperactivity disorder, depression, and anxiety (15, 16), and half of all mental health disorders occur during adolescence (16). Demands and competitive pressures are placed on adolescent athletes to perform at high standards in organized sports, while adolescent athletes also experience stressful developmental periods during puberty (17). Adolescent athletes experience frequent mood swings and fluctuating training performance (17–19). Adolescent athletes also experience fatigue from intensive training conducted almost year-round (20), and stress negatively impacts their physical and mental conditions (21, 22), which in turn can lead to injuries (22–24). Young athletes are often less prepared to face challenging issues in organized or competitive sports (25, 26). As a result, many young athletes choose to drop out of competitive sports and opt not to continue their participation.

Research has shown that adolescent athletes have various positive and negative impacts from their participation, but the contribution of social support is often overlooked. How do adolescent athletes interpret their experiences in competitive sports activities in their development? The research findings indicate that the experiences of adolescent athletes are influenced by support from their social networks, such as demonstrating resilience during difficult times (25, 27, 28). Therefore, adolescent athletes need to receive adequate support in their sports and from the social networks around them to minimize the negative impact on their long-term health, well-being, and performance (29, 30). Participation in sports contributes to self-confidence through encouragement, self-talk, and successful performance (31, 32). Thus, successful performance at the elite level inevitably develops athletes’ self-confidence and their ability to manage emotions under pressure, influenced by their social environment (32). However, much of the literature on youth sports highlights the reasons for discontinuing training in competitive sports (33–36) from various perspectives of social support.

Thus, although several reviews broadly discuss this area, for example (37–40), to our knowledge, a review specifically focusing on adolescent athletes exploring experiences qualitatively and influencing their development in competitive sports is needed by synthesizing and uncovering the essence of the existing literature. This article presents the results of a systematic review that investigates the significant experiences of adolescent athletes in competitive sports activities in their development. Then, the information obtained from the systematic review is used to develop a conceptual model of adolescent athletes in competitive sports life. The conclusions of our review will be directly relevant to those working in competitive sports environments for adolescents.

## 2 Methods

The research utilized a systematic literature review method, as outlined in study (41). This approach followed the principles of a systematic literature review with a descriptive qualitative design, aligned with the research questions, to explore the body of publications on a specific topic based on existing literature.

The search strategy was developed through consultation among co-authors with expertise in youth sports. H.P.R, a seasoned journal editor with expertise in systematic searches, took the lead in performing searches across databases and organizing the articles for review. The initial online search was carried out in April 2023 and updated on January 2025, using electronic databases such as Web of Science (WoS), PubMed, and Scopus, which are among the most comprehensive academic search engines (42). The article search process followed the Preferred Reporting Items for Systematic Reviews and Meta-Analyses (PRISMA) guidelines (43, 44). Furthermore, the findings in each article were evaluated and interpreted (45).

The purpose of this research was to understand how adolescent athletes interpret their experiences in competitive sports, including psychological, social, and cultural aspects. Therefore, the search equation was designed to encompass concepts such as the meaning of experience, the identity of adolescent athletes, and the impact of competitive sports. Several key terms relevant to the research theme include: (a) Adolescent athlete: “youth athlete,” “adolescent athlete,” “young athlete,” “junior” (b) Meaning of experience: “meaning,” “perception,” “experience,” “identity” (c) Competitive sports: “competitive sports,” “elite sports,” “high-performance sports,” “Sport Achievement”. Search queries used Boolean operators such as “AND” and “OR” to ensure the search results were relevant and specific. These search terms included: (“youth athlete” OR “adolescent athlete” OR “young athlete” OR “junior”) AND (“meaning” OR “perception” OR “experience” OR “identity” OR “life”) AND (“competitive sports” OR “elite sports” OR “high-performance sports” OR “sports achievement”).

The design followed the PICOS strategy, which defined participants, intervention, outcome, and study design. We defined the inclusion and exclusion criteria in the PICOS table, which can be seen in (Table 1). The research results chapter grouped all article findings relevant to the research theme, namely adolescent athletes in competitive sports, into specific field categories.

**Table 1:** PICOS.

Parameter	Inclusion	Exclusion
Population	Athletes	Coach, parents, staff, and referee
Intervention	Athletes participate in an organized and competitive sports environment.	Sports aimed at health, sports as a hobby, gathering, or recreation, sports in the form of traditional games.
Outcomes	Experience of being a adolescent athlete. Experience as a adolescent athlete. Meaningful experiences about psychological and social identity.	Experience of being a professional athlete. Experience of being an athlete in adulthood. Study results on physiological aspects, fitness, study results on biomechanics, and focusing on economic, political, or legal aspects in sports.
Study design	Studies using qualitative methods or mixed methods with a qualitative focus.	Quantitative analysis methods or techniques.
Restrictions	The publication period of the articles is from 2014 to 2024.	Articles published before the year 2014.

Source: own elaboration.

### 2.1 Study selection

Studies retrieved from each database were initially exported to Microsoft Excel. Duplications were carefully checked and flagged for exclusion on subsequent Excel tab sheets. Two authors (H.P.R. and A.K.) were tasked with independently selecting studies by reading the title and abstract, followed by the full text, and identifying systematic reviews that met the eligibility criteria. Discrepancies regarding study inclusion were discussed and resolved by consensus with the author (M.F.D.). A manual search in the references of the selected articles used the same eligibility criteria as the studies obtained from the electronic database search.

### 2.2 Data extraction

The initial search using Boolean operators and relevant keywords across electronic databases yielded a total of 367 articles. Two authors, H.P.R. and A.K., extracted data based on key characteristics of each study. They organized the information into columns: author, publication year, article title, journal, database searched, study purpose, and methodology. The extraction process was carried out methodically and carefully. In the end, 19 articles were selected that met the inclusion criteria. A comprehensive summary of the study characteristics, including sample details, research methods, and key findings, is provided in (Table 2).

**Table 2:** Characteristics of the included studies.

Study Author, year (ID)	Research design	Sample characteristics	Type of sport	Research objectives	Important findings
Lisinskiene et al., 2018 (1) (50)	Mixed-methods	15 adolescent athletes (age between 15 and 16 years)	Team sport and individual sport	The purpose of this study was to examine the relationship between participation in youth sports and youth-parent attachment.	Discovering adolescent athletes’ expressions in competitive sports, their relationships with coaches and parents in competitive sports, and the consequences they face.
Pérez et al., 2022 (2) (55)	Qualitative method	45 former female athletes (experiences during adolescence 13–20 years)	Futsal Handball Volleyball Track and field Swimming Fencing	The purpose of the study was to analyses women's perceived barriers to continued participation in competitive sport.	Her findings on the various factors that act as barriers to women in competitive sports.
Thomas et al., 2019 (3) (39)	Qualitative method	16 former world-class track and field athletes (experiences during adolescence 13–20 years)	Track and field	The purpose of the study was to explore through a socio-cultural lens the perceived early training and competition environment, and support network and progression during early childhood and adolescence.	The findings are about the sport environment influencing the development of athletes at a young age: Conducive sports environment, functional social support network, contribution from key organizations.
Thomas et al., 2021 (4) (38)	Qualitative method	11 former junior-elite (experiences during junior athletes under 17 years)	Track and field	The purpose of the study was to explore the underlying motives that may have contributed to the unsuccessful transition and subsequent dropout.	Key factors identified as barriers to transition: (1) ‘not enough support’; (2) ‘feeling pressurized to make sure I commit’; (3) ‘it's always competitive here’; and (4) ‘battling injuries.’
Neely et al., 2017 (5) (49)	Qualitative method	14 female adolescent athletes (average age of athletes 15 years)	Soccer, Basketball Volleyball Ice Hockey	The purpose of the study was to look at how female adolescent athletes and their parents cope with being excluded from competitive sporting events.	The participants viewed the deselection experience from the same perspective (i.e., athletes and parents viewed deselection as ‘our problem’), and the responsibility for coping with deselection changed over time. Athletes and parents then engaged in cooperative actions to manage their reactions to the stressor (the ‘our problem, our responsibility’ orientation).
White and Bennie 2015 (6) (40)	Qualitative method	22 adolescent athletes (age between 10 and 16 years old)	Gymnastic	The purpose of this study was to investigate gymnast and coach perceptions about the development of resilience through gymnastics participation.	The findings of this study reveal aspects of the gymnastics environment created stress and exposed gymnasts to many challenges in training and competition. Features of the sport environment, such as interpersonal relationships and positive coach behaviors, supported gymnasts through these challenges and encouraged them to overcome failure. Gymnastics participation was perceived to develop resilience, as well as life skills, self-efficacy, and self-esteem.
Elliott et al., 2017 (7) (53)	Qualitative method	50 male athletes (average age 15 years)	Football	The aim of this study was to understand the experiences of being a TI youth athlete and present the findings as “lessons” for parents seeking to enhance their involvement in TI (talent-identified) youth sport settings.	The thematic analysis identified three major themes from the focus groups with TI youth athletes: (a) Difficulties with talent, (b) Navigating the future, and (c) Playing for improvement. The findings offer a number of lessons for parents and youth sport organizations to facilitate the transfer of knowledge to an applied setting.
Parent et al., 2014 (8) (61)	Mixed-methods	55 adolescent athletes (age between 14 and 18 years old)	Nordic Ski Snow-Board Freestyle Skiing Curling Ice Hockey Speed Skating	The purpose of this article was to understand young athletes’ experiences at a youth sport festival, specifically the Youth Olympic Games (YOG).	The research findings describe YOG athletes, including feeling positive and negative things during the implementation.
Strachan and Davies et al., 2014 (9) (52)	Qualitative method	26 adolescent athletes (age between 13 and 18 years old)	Swimming Gymnastics and variety of sport.	The purpose of the study was to use photo elicitation to explore youth experiences and positive development in sport.	The youth sport experiences varied between the two contexts. Features related to supportive relationships were more prominent in the sports camp context, while the high-performance context reported more features related to skill development opportunities.
Jacobsson et al., 2018 (10) (54)	Qualitative method	74 participant (24 adolescent athletes)	Track and field	The aim of this study was to establish what is perceived to contribute and cause injuries in youth track and field by compiling the best available experiential knowledge about the underlying factors and use this knowledge to identify appropriate areas to handle these in practical ways.	Injuries in young athletes are not considered to be the result of individual factors alone but rather the result of an interaction between multiple factors at various levels. Three main factors emerged as follows: There is a deficiency of understanding about athletic development in daily training, a lack of inclusivity in training communities and sport policies, and a lack of public health practices.
McCalpin et al., 2016 (11) (48)	Qualitative method	17 adolescent athletes (age between 13 and 18 years old)	Football	The purpose of this study was to explore young female athletes’ experiences in their modified soccer environment.	The athletes perceived their competitively engineered soccer experience as a distinct environment that emphasized personal development, positive relationships, and the underlying enjoyment of sport, according to the results. These findings shed light on how youth sport structure modifications influence the athletes’ experiences, providing practical implications to further promote positive youth sport experiences.
Larson et al., 2018 (12) (59)	Qualitative method	20 former swimming athletes (experiences during junior athletes under 17 years)	Swimming	The purpose of this study was to explore how youth swimming experiences shape subsequent participation in masters swimming.	Analyses revealed disruptive, attractive, and enabling forces stemming from youth swimming. For recreational swimmers, high training volume in youth swimming led to negative physical and emotional consequences and scheduling conflicts between swimming and other activities. When they felt their performance was no longer improving, these costs led them to drop out of youth swimming. In contrast, the continuers’ focus on enjoyment, social aspects, and other non-performance-related reasons for swimming led to a smooth transition from youth swimming into masters swimming. As adults, rekindles had confidence in their swimming abilities because of their youth training, but they required a shift away from performance-related motives in order to return to swimming.
Mallinson-Howard et al., 2018 (13) (51)	Mixed-methods	19 female athlete (age between 13 and 18 years old)	The various types of sports available in the school's extracurricular program and club sports	The purpose of the study was to demonstrates that four subtypes of perfectionism from the 2 × 2 model are associated with different youth sport experiences. This study provided the first exploration of the experiences of youth sport participants exhibiting different subtypes of perfectionism using mixed-methods.	The meanings that youth sport participants gave to their involvement in sport (i.e., goals, values, and intentions) and the features of the social environment that they considered important differed across the four perfectionism subtypes. Participants from all four subtypes described the importance of coaches and peers, with some groups identifying different roles for coaches in terms of type and amount of involvement.
Timpka et al., 2021 (14) (2)	Qualitative method	14 adolescent athletes (under 17 years old)	Athletics	This study aimed to investigate experiences of medical service provision among high-level adolescent athletics (track and field) athletes from three continents.	The young athletes experienced health problems in various places, from their homes and local sports facilities in rural areas to competition venues and boarding schools. The athletes recounted a wide range of health problems. Many of the athletes reported gradually developing injury pain during training, hamstring muscle strain, and ankle distortion. Regarding illnesses, athletes mostly reported acute illnesses, such as influenza, chickenpox, and appendicitis. Some athletes suffered from long-term illnesses such as eating disorders, malaria, asthma, and mood disorders. The athletes received varying levels of medical support.
Ronkainen et al., 2022 (15) (3)	Qualitative method	16 national athletes (age between 19 and 20 years old)	Team sport and individual sport	The purpose of this study was to expanded traditional understandings of sport-based youth development by exploring existential learning in sport through encounters with discontinuity.	Themes His findings on progress and setbacks include facing possibilities and limitations, being yourself and others, and living the life of an elite athlete.
Bean et al., 2020 (16) (60)	Mixed-methods	47 adolescent athletes (average age 14 years)	Hockey Volleyball	The purpose of this study was to explore the processes of life skills development and transfer in youth hockey and volleyball.	His findings about life skills Learning psychosocial skills Sport aids in the development of certain skills, such as respect and communication. Sport aids in the development of confidence, life skills, and knowledge. Improving leadership qualities through sport participation Sport participation fosters the development of both structured and unstructured leadership roles.
Battaglia et al. 2019 (17) (80)	Qualitative method	32 participants (15 adolescent athletes) (age between 11 and 19 years old)	Team sport and individual sport	The purpose of this study therefore was to examine athletes’, parents’, and coaches’ interpretations of the term dropout from youth sport through qualitative inquiry.	Participants associated dropout with failure: a lack of commitment, unrealized athletic potential, and inappropriate reasons for leaving. Participants suggested the term dropout lacked relevance in sport, citing the voluntary nature of sport and leaving as developmentally appropriate transitions.
Littlefair and Nichol 2019 (18) (58)	Qualitative method	18 participants (10 adolescent athletes)	Cricket	The aim of this study is to gain an insight into the experiences of children and stakeholder adults in such a setting.	This research highlights the excellent development and learning environment within youth cricket clubs and alludes to gaps within the wider field of youth sport. The three selected groups, the players, parents, and coaches, have successfully aligned themselves to create a supportive and non-threatening environment to enable children to learn and develop their confidence, self-esteem, and skills.
Koulanova et al. 2021 (19) (56)	Qualitative method	71 participants (20 girl athletes, ages 13–18 years old)	Team sport (water polo, hockey, rugby, volleyball, basketball, soccer, and softball)	The aim of this qualitative study is to identify feasible and realistic strategies to reduce and address body image concerns among adolescent girls involved in team sports at recreational or competitive levels.	Actionable strategies were identified that encompass four main themes: eliminating body image stigma, rethinking uniforms and sportswear, top-down—everyone has a role, and modeling positive body roles. These strategies encompass various systemic, environmental, social, and individual levels operationalized within an ecological model.

Source: own elaboration.

These articles were then subjected to further synthesis in order to facilitate an investigation of the pivotal findings emanating from the research. In the subsequent analysis, we identified significant findings that would guide the researcher in developing the research theme, as illustrated in the table below: (i) Author, year (ii) Sample characteristics (iii) Type of sport, (iv) Research objectives (v) Research design (vi) Important findings (vii) Research themes. Data was input according to the table columns provided.

The initial step in determining the theme of each article was based on its main findings. These findings were carefully reviewed multiple times until the content was fully understood. The interpretation of these findings was then used to derive a major theme of the article. At this stage, the themes were not categorized or grouped but were formed freely based on the author's in-depth understanding. Subsequently, the main themes of the articles were organized into similar categories, leading to three research themes: obstacles, meaning, and development. These themes were color-coded (yellow for meaning, red for barriers, green for athlete development) to aid in the grouping process.

We then synthesized the key research findings from each article within the same thematic group. At this stage, we simplified the key findings and provided conclusions for each article. After that, we grouped each simplified conclusion into a table with intersections or closeness of meaning. Finally, we pulled the key findings back into the subordinate themes and gave them meaning and appropriate descriptions. For a clearer understanding of the data synthesis process, see an example of the analysis of the theme The meaning of competitive sports for adolescents (Table 3).

**Table 3:** Theme synthesis process the meaning of competitive sports for adolescent.

Simplified results research findings ID 1, 4, 5, 9, 11, 13	Simplification group	Thema superordinate important findings
• The heavy burden of being a gifted athlete • Building Identity • Exciting, yet constantly on the Rise • Importance of physical and psychological safety • Supportive relationships • Building confidence and persistence, motivation, and gaining recognition • Positive social norms • Developing skills • Opportunity to belong • Emphasizing Joy • Strong Friendships • Constantly Evolving Challenges • Unique and potentially positive (including relationships with family). • Sport A fun hobby for friendship and learning. • Collaborative to achieve team success • A sense of belonging, togetherness and hiding in the crowd. • Time to shine and assert self-worth.	Fun participation Life Coach Losing a Friend	Fun that features Progressive • Fun participation • `Fun, yet constantly in Improvement • Sports A fun hobby for friendship and learning • Emphasizes Fun • Positive social norms • These themes relate to pride, fun, responsibility, positive values, and commitment/dedication. • Importance of physical and psychological safety
	Importance of physical and psychological safety Supportive relationships Building confidence and persistence, motivation, and gaining recognition Positive social norms (pride, fun, responsibility, positive values, and commitment/dedication). Developing skills Opportunity to belong Emphasis on Fun Strong Friendships Constantly Evolving Challenges Unique and potentially positive (including relationships with family).	A sense of community that supports each other • Opportunity to have • A sense of belonging, togetherness, and hiding in the crowd. • Strong Friendships • Life Coach (second parent) • collaborative to achieve team success • Supportive relationships • Build confidence and persistence, motivation, and gain recognition • Unique and potentially positive (including relationships with family).
	Sport a fun hobby for friendship and learning. Collaborative to achieve team success A sense of belonging, togetherness and hiding in the crowd. A time to shine and assert self-esteem.	Consequences and challenges continue to evolve • Evolving Challenges • Athletes to feel that they are being broadly challenged, especially increasing the importance of challenges that go beyond just scoring goals. • Developing skills • Skills, including knowing the rules, physical skill development, knowledge relating to new strategies and skills, and goal setting • The heavy burden of being a gifted athlete • Building Identity • Time to shine and assert self-esteem. • Losing Friends

Source: own elaboration.

### 2.3 Risk of bias assessment (ROBIS)

The risk of bias and methodological quality of the included studies were assessed independently by two authors with an understanding of adolescent athletes (C.S. and H.S.) using Risk of Bias Assessments. Disagreements between reviewers were resolved through discussion with a third reviewer (A.K). We adopted the risk of bias assessment for the study, a systematic review of talent assessment in sports (46), and the methodological quality of the included studies using the critical appraisal for qualitative studies (containing 21 items) (47). Each qualitative article is evaluated based on the following 21 items: objectives (item 1), literature review (item 2), study design (items 3, 4, and 5), sampling (items 6, 7, 8, and 9), data collection (descriptive clarity: items 10, 11, and 12; procedural rigor: item 13), data analysis (analytical rigor: items 14 and 15; audibility: items 16 and 17; theoretical connection: item 18), overall rigor (item 19), and conclusion/implications (items 20 and 21). The results per item are coded as 1 (meets the criteria), 0 (does not fully meet the criteria), or NA (not applicable). The final score expressed in relative value is reported for each study following the assessment guidelines (46). The final score is the total of all scores for specific items divided by the total number of items assessed for that specific research project (i.e., 21 items). Classification of articles as (1) low methodological quality - with a score <50%; (2) good methodological quality—score between 51% and 75%; and (3) very good methodological quality—with a score > 75%.

## 3 Results

### 3.1 Study selection

The research team examined an initial set of 367 articles generated by the search engine: Web of Science (WoS) = 158, Scopus = 182, PubMed = 27. The initial screening identified 55 disqualified articles consisting of duplicate files, resulting in 312 articles to be re-selected. From the 312 articles, our team conducted a re-screening by examining the relevance of the title to the research topic, the article's completeness, the year of publication, and the article's eligibility. The result was that 222 were excluded, leaving 90 articles included. The report was assessed for eligibility, and 90 articles met the criteria for full-text analysis. All important information from the articles in Microsoft Excel includes the author, publication year, title, journal, objective, method, participants, research objective, and results.

The result was that 71 articles were excluded for the following reasons; twelve (12) articles have participants who have reached adulthood, as many as twelve (12) articles use quantitative methods or questionnaires and the like that do not capture the meaning and experience of participants, as many as twelve (12) articles discuss health sports, recreation, and traditional games, fifteen (15) of his research articles focus on health, biomechanics, and physical, twenty (20) articles do not focus on exploring teenagers’ experiences or being adolescent athletes.

**Figure 1:**
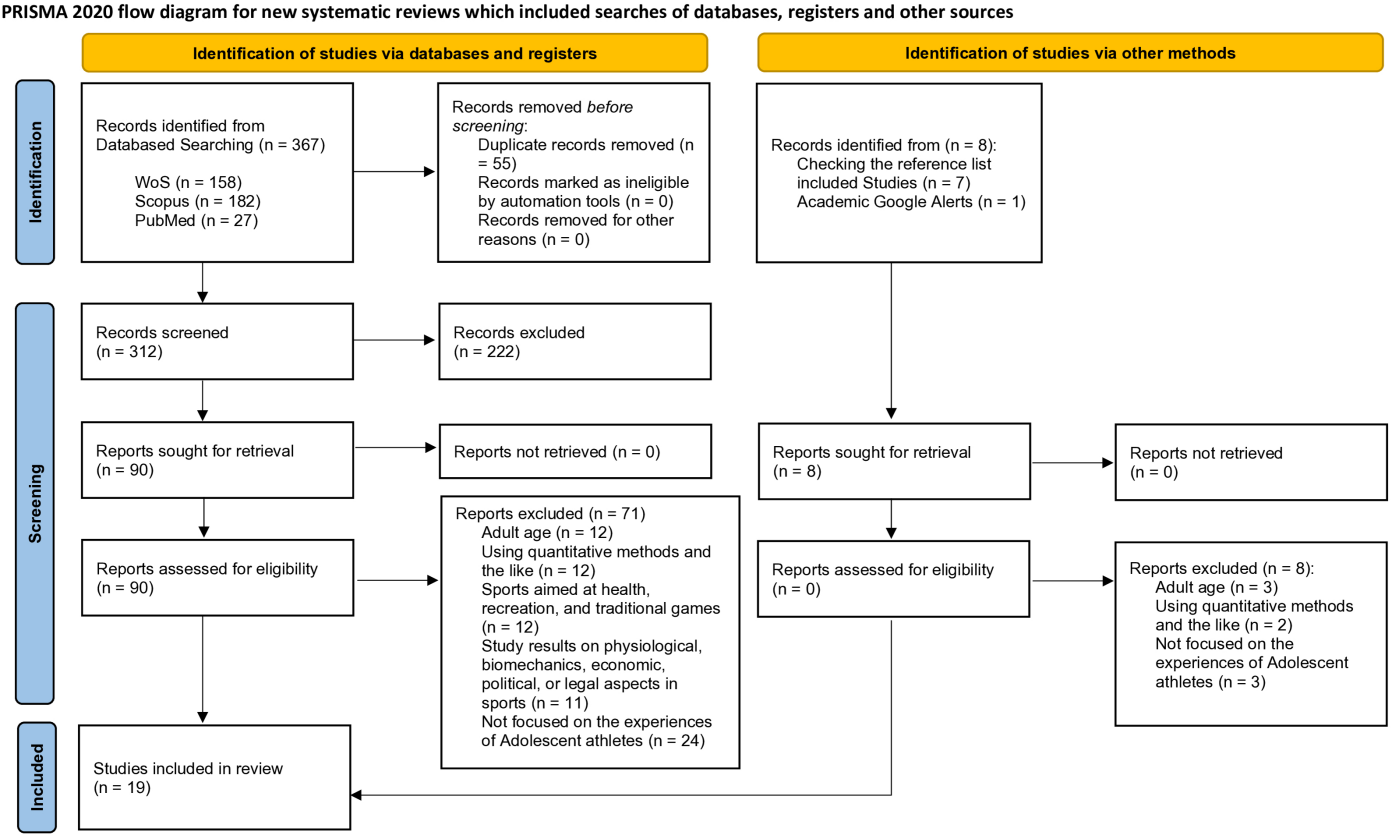
Flowchart illustrating the screening process. PRISMA 2020 flow diagram showing number of reports collected and number of eligible studies after the screening process.

From a rigorous selection process, 19 articles were included. All included articles were eligible for analysis and synthesis as they met the criteria of: having adolescent participants, using qualitative methods with an emphasis on meaning and immersive experiences, including competitive and organized sports, research findings on the psychological and social environment of being an adolescent athlete, and lastly focusing on the experience of being an adolescent athlete or during being an adolescent athlete. The PRISMA flowchart summarizes the details of all study options (Figure 1).

### 3.2 Descriptive results

**Figure 2:**
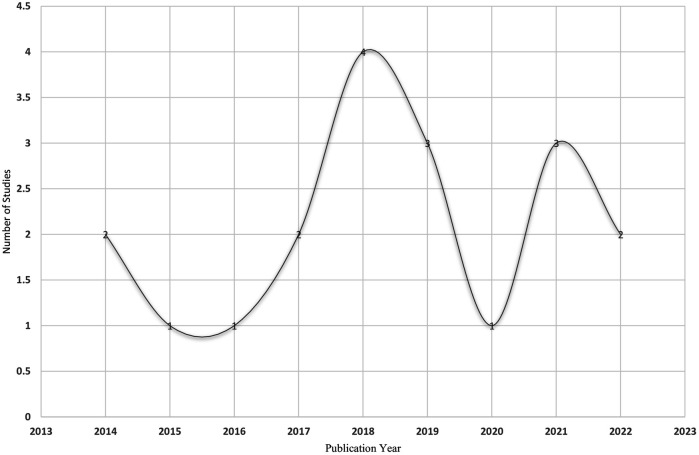
The number of studies on significant experiences of adolescent athletes based on the year of publication.

The results risk of bias assessment of the research methodology quality of the 19 included qualitative studies is as follows: (1) the average score for the 19 included qualitative studies is 96.0%; (2) 9 articles achieved a maximum score of 100%; (3) no publication received a score below 75%. The results of the bias risk assessment of the included review are presented in (Table 4). Figure 2 illustrates the publication year profile of the studies. Of the included studies, 13 (68.4%) were published within the 5 years between 2018 and 2023. The remaining publications on the experiences of adolescent athletes in competitive sports consistently only range from 1 study (2015, 2016) to 2 studies (2014, 2007).

**Table 4:** Risk of bias in included studies of the systematic reviews .

Authors, year, ID	Q1	Q2	Q3	Q4	Q5	Q6	Q7	Q8	Q9	Q10	Q11	Q12	Q13	Q14	Q15	Q16	Q17	Q18	Q19	Q20	Q21	Score
Lisinskiene et al., 2018 (1) (50)	1	1	1	1	1	1	1	1	1	1	1	1	1	1	1	1	1	1	1	1	1	100%
Pérez et al., 2022 (2) (55)	1	1	1	1	1	1	1	1	1	1	0	1	0	1	0	1	1	1	1	1	1	85%
Thomas et al., 2019 (3) (39)	1	1	1	1	1	1	1	0	1	1	1	1	1	1	1	1	1	1	1	1	1	95%
Thomas et al., 2019 (4) (38)	1	1	1	1	1	1	1	1	0	1	1	1	1	1	0	1	1	1	1	1	1	90%
Neely et al., 2017 (5) (49)	1	1	1	1	1	1	1	1	1	1	1	1	1	0	1	1	1	1	1	1	1	95%
White and Bennie 2015 (6) (40)	1	1	1	1	1	1	1	1	1	1	1	1	1	1	1	1	1	1	1	1	1	100%
Elliott et al., 2017 (7) (53)	1	1	1	1	1	1	1	1	1	1	1	1	1	1	1	1	1	1	1	1	1	100%
Paren et al., 2014 (8) (61)	1	1	1	1	1	1	1	1	1	1	1	1	1	1	0	1	1	1	1	1	1	95%
Strachan and Davies et al., 2014 (9) (52)	1	1	1	1	1	1	1	1	1	1	1	1	1	1	1	1	1	1	1	1	1	100%
Jacobsson et al., 2018 (10) (54)	1	1	1	1	1	1	1	1	1	1	1	1	1	1	1	1	1	1	1	1	1	100%
McCalpin et al., 2016 (11) (48)	1	1	1	1	1	1	1	1	1	1	0	1	1	1	1	1	1	1	1	1	1	95%
Larson et al., 2018 (12) (59)	1	1	1	1	1	1	1	1	1	1	1	1	1	1	1	1	1	1	1	1	1	100%
Mallinson-Howard et al., 2018 (13) (51)	1	1	1	1	1	1	1	1	1	1	1	1	1	1	1	1	1	1	1	1	1	100%
Timpka et al., 2021 (14) (2)	1	1	1	1	1	1	1	1	1	1	1	1	1	1	1	1	1	1	1	1	1	100%
Ronkainen et al., 2022 (15) (3)	1	1	1	1	1	1	1	1	1	1	0	1	1	0	1	0	1	1	1	1	1	85%
Bean et al., 2020 (16) (60)	1	1	1	1	1	1	1	1	1	1	1	1	1	1	0	1	1	1	1	1	1	95%
Battaglia et al. 2019 (17) (80)	1	1	1	1	1	1	1	1	1	1	1	1	1	1	1	1	1	1	1	1	1	100%
Littlefair and Nichol 2019 (18) (58)	1	1	1	1	1	1	1	1	1	1	1	0	1	1	1	1	1	1	1	1	1	95%
Koulanova et al 2021 (19) (56)	1	1	1	1	1	1	1	1	1	1	1	0	1	1	1	0	1	1	1	1	1	90%

Authors, Year, ID: Author's name, year of publication, and Article ID. Q1–Q21: Research quality evaluation questions (see the list below). 1 for studies that meet the criteria or answer “Yes,” 0 for studies that do not meet the criteria or answer “No.” Score: The total percentage of “1” (positive) answers against the total number of questions.

**Question List (Q1**–**Q21).**

1. Are the research objectives and/or research questions clearly stated?

2. Has a relevant literature review been conducted?

3. Is the research design aligned with the research objectives?

4. How is the data collection method explained in this research?

5. Were the participants explained in sufficient detail?

6. How is the participant recruitment process carried out?

7. Is there any information regarding research ethics, including participant consent?

8. Is the research environment clearly explained?

9. How is the data collected (for example, interviews, observations, documents)?

10. Is the data analysis method explained in detail?

11. How did the authors ensure the trustworthiness of the data?

12. Are the research results presented in sufficient depth?

13. Are there direct quotes from participants in the research results?

14. How is the relationship between the findings and the literature review used?

15. Is there a critical reflection on the potential bias in the research?

16. Are the implications of the research for practice or policy discussed?

17. How are the limitations of this research identified and explained?

18. Is the conclusion supported by the presented findings?

19. How clear is the reporting of this research (structure and language)?

20. Are there any recommendations for further research?

21. Does this research have a significant contribution in its field?

As shown in the data description results (Table 5), this study is specifically aimed at examining the experiences of adolescent athletes in competitive sports life, with participants generally under 18 years old, totaling 14 (74%). In contrast, 5 (26%) adult participants are former athletes reflecting on their experiences as adolescent athletes. Because the emphasis is on the experiences and meanings encountered, this study predominantly used qualitative methods, with 15 (79%) employing qualitative research and 4 (21%) using a mixed-method approach. All the sports included are organized competitive sports with a wide variety of types; there are 6 (31.6%) team or group types, 6 (31.6%) individual types, and 7 (36.8%) a mix of individual and group types. This research yields significant experiences of adolescent athletes in competitive sports activities in their development through literature that includes (19 studies), divided into three themes. First, a depiction of the most meaningful experiences provided by adolescent athletes at every moment of their journey in competitive sports was presented in a study of 6 (31.5%). Second, a review of negative experiences that hindered adolescent athletes in competitive sports was examined by several 6 studies (31.5%). Third, positive experiences that influence athlete development were reviewed with a total of 8 studies (37%).

**Table 5:** Descriptive statistics.

Age	*N* (%)
Youth (6–11)	0
Adolescent (12–17)	14 (74%)
Adult (18+)	5 (26%)
Study design	
Qualitative method	15 (79%)
Mixed-methods	4 (21%)
Type of sport	
Team sport	6 (31.6%)
Individual sport	6 (31.6%)
Mixed	7 (36.8%)
Findings theme	
Meaningful experiences	6 (31.5%)
Barriers to athlete development	6 (31.5%)
That influence athlete development	7 (37%)

Source: own elaboration.

Table 6 summarizes the synthesis of the research theme findings. The theme of meaningful experiences, in terms of enjoyment, shows progression (48–51) 67% (4/6) studies show that participation in competitive sports is considered enjoyable, exciting, pride-inducing, skill-enhancing, and continuously improving. Regarding the sense of community that supports each other (48–52) 83% (5/6) studies discuss that adolescent athletes experience a sense of togetherness (parents, coaches, friends), which in turn forms a community that creates mutually supportive relationships for the athletes’ progress. In terms of consequences (48, 50, 53) 50% (3/6) studies report that loss of friends, frequency of play, and high social expectations become the consequences and challenges that adolescent athletes bear to continue developing. On the theme of barriers for adolescent athletes: in terms of prolonged injuries (2, 54, 55) 50% (3/6) studies report injuries as a highly negative experience that significantly affects their participation in competitive sports. Regarding weak commitment (38, 55–57) 67% (4/6) studies report pressure from various sources affecting the commitment of adolescent athletes to participate in competitive sports. In terms of an unsupportive sports environment (38, 54, 56) 50% (3/6) studies report various inadequate support such as funding, transparency, and psychological support for adolescent athletes. On the theme of the influence of athlete development: in terms of a constructive training atmosphere (39, 40, 58, 59) 57% (4/7) studies describe how athletes feel that a well-established training atmosphere makes them feel enjoyable, relaxed, and easy to socialize, enjoying the social aspect as members of the sports community. In terms of Connectedness 43% (3, 39, 58) (3/7), studies report that the connections formed (family, friends, coaches) create stability for adolescent athletes, which in turn positively influences their development. In terms of personality development (3, 39, 60, 61) 57% (4/7), studies report that positive experiences in competitive sports have a transformative impact on the personal development of adolescent athletes, such as discipline, confidence, leadership, and socio-cultural skills valuable for their future.

**Table 6:** The synthesis results of the research theme on the experiences of adolescent athletes in competitive sports.

Synthesis	*N*	Percentage (%)	Study
Meaningful experiences			
Fun featuring progressive	(4/6)	67%	McCalpin, Evans, and Côté 2017; Neely et al. 2017; Lisinskiene, Guetterman, and Sukys 2018; Mallinson-Howard et al. 2018)
A sense of community that supports	(5/6)	83%	McCalpin, Evans, and Côté 2017; Neely et al. 2017; Mallinson-Howard et al. 2018; Strachan and Davies 2015; Lisinskiene, Guetterman, and Sukys 2018
Consequences and challenges	(3/6)	50%	McCalpin, Evans, and Côté 2017; Elliott, Drummond, and Knight 2018; Lisinskiene, Guetterman, and Sukys 2018
Barriers to athlete development			
Prolonged injury	(3/6)	50%	Jacobsson et al. 2018; Pérez, Giménez, and Posadillo 2022; Timpka et al. 2021
Weak commitment	(4/6)	67%	Koulanova et al. 2021; Thomas et al. 2021; Battaglia and Kerr 2022; Pérez, Giménez, and Posadillo 2022
Unsupportive sports environment	(3/6)	50%	Thomas et al. 2021; Jacobsson et al. 2018; Koulanova et al. 2021
That influence athlete development			
Constructive training atmosphere	(4/7)	57%	Littlefair and Nichol 2019; White and Bennie 2015; Thomas et al. 2019; Larson et al. 2019
Interconnectedness	(3/7)	43%	Ronkainen et al. 2023; Thomas et al. 2019; Littlefair and Nichol 2019
Personality development	(4/7)	57%	Thomas et al. 2019; Bean, Kramers, and Harlow 2022; Ronkainen et al. 2023; Parent, Kristiansen, and Macintosh 2014

**Figure 3:**
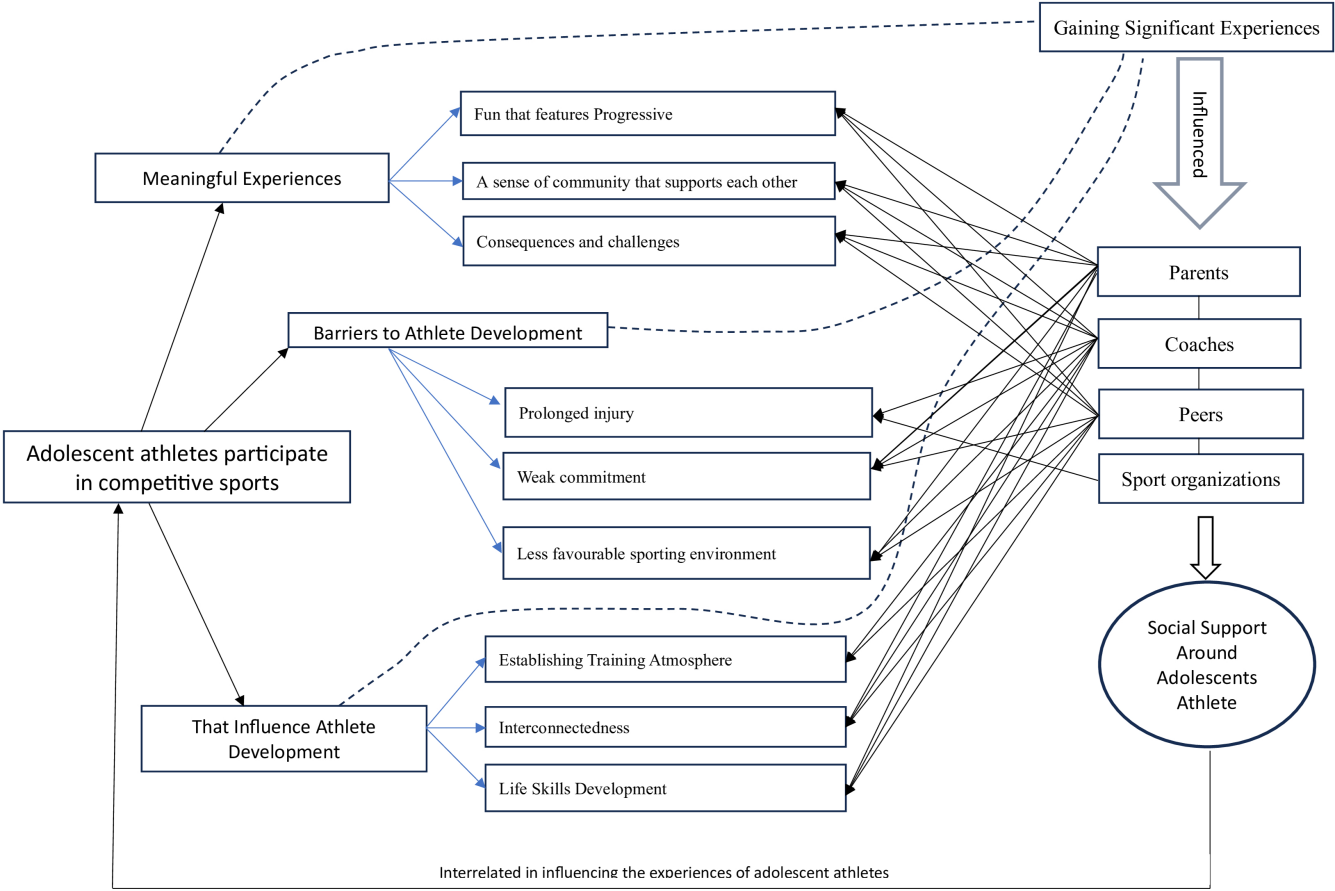
The pattern of research findings on the essence of adolescent athletes' participation with social support.

Based on the findings from the synthesis of each theme of the included research results, the concept of the social environment influencing the significant experiences of adolescent athletes in competitive sports has been developed and presented visually in (Figure 3). This concept explains that in competitive sports activities, adolescent athletes gain meaningful experiences, experiences that become obstacles, and experiences that influence their development. The capture of meaningful experiences refers to adolescent athletes who attribute deep significance to every moment of their journey in organized sports. Experiences that become obstacles lead to the challenges or hurdles athletes face when participating in competitive sports. Developmental experiences occur through a reciprocal process of competitive sports activities for adolescent athletes, influenced by various factors that affect the progressive changes in adolescent athletes. This conceptual model then views the significant experiences of these adolescent athletes as a process of their activities in competitive sports, with the surrounding social support interactions being highly influential. Adolescent athletes consistently mention coaches, parents/family, friends, and organizations influencing their journey in competitive sports, ultimately creating significant experiences for them. Coaches, for example, are almost involved in all significant experiences for adolescent athletes; meaningful experiences can be created through enjoyable training patterns (50), the sense of togetherness among coaches like family (52), and the coaches’ art of continuously challenging athletes (48). Even though it can have negative meanings that become obstacles, such as the wrong training patterns (54), the coach's anticipation affecting commitment (56), and the lack of attention from the coach (57). The coach is someone who influences the development of athletes by creating a training atmosphere (39), the positive behavior of the coach (40), and providing a strong personality (60).

## 4 Discussion

**Figure 4:**
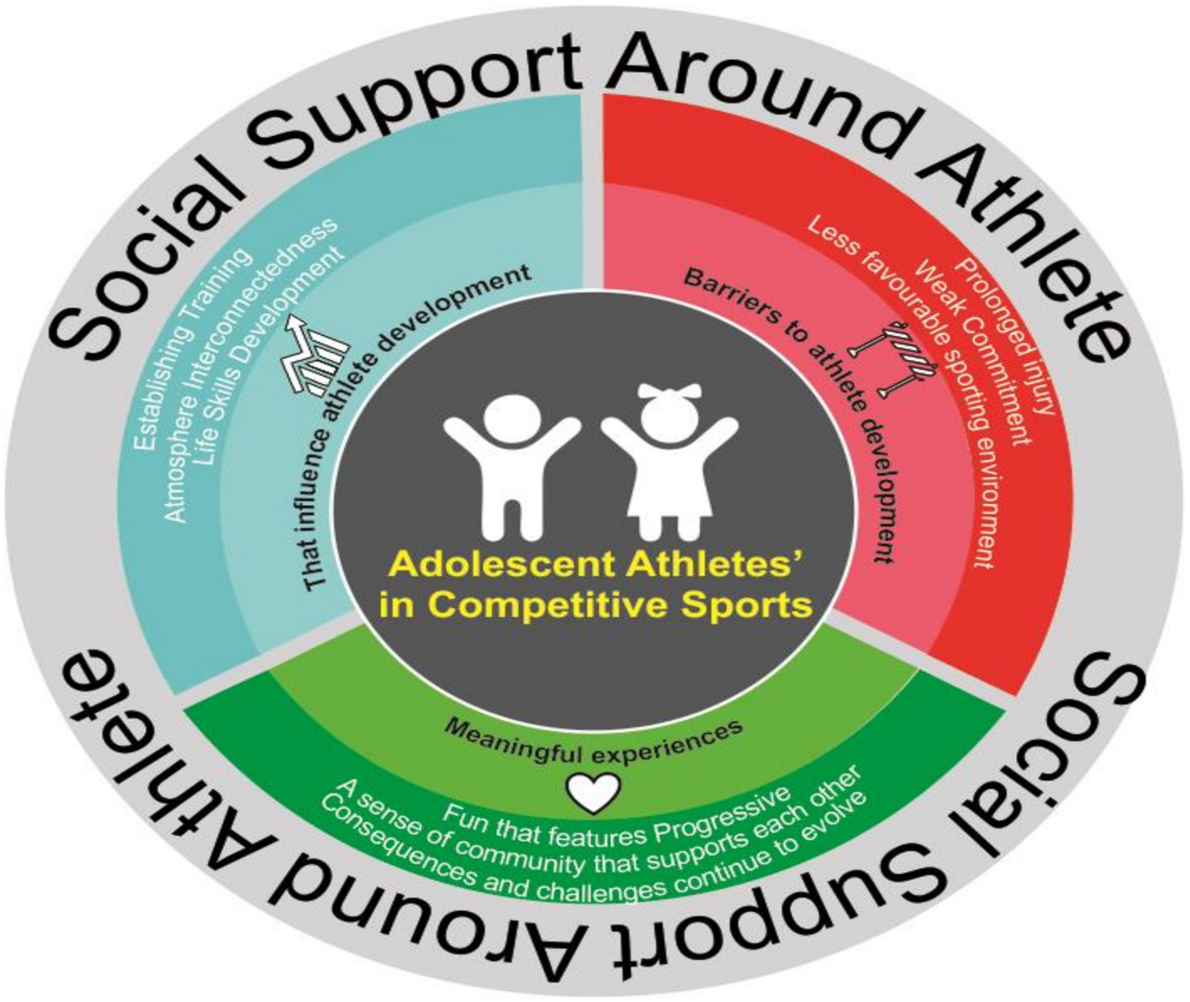
Flowchart illustrating important experiences of adolescent athletes in competitive sports.

This systematic review investigates the scientific evidence regarding the significant experiences of adolescent athletes’ participation in competitive sports life. This is the first systematic review highlighting key aspects of adolescent life in the complex realm of competitive sports, based on evidence from 19 studies representing the lives of adolescent athletes with a qualitative approach. Based on the analysis and synthesis of the literature included in this review, the experiences of adolescent athletes participating in competitive sports life are depicted (Figure 4). The following section will discuss some of the most interesting findings from this review.

### 4.1 Meaningful experiences in the competitive sports life of adolescent athletes

Adolescent athletes in competitive sports must create much excitement in the early stages of participation. Pleasure is an important predictor of athletes’ intrinsic motivation in the conclusion of his research (62). There is a sense of fulfillment within them because the competitive element of sports is closely tied to competition, pride, showcasing developing skills, and winning medals, but enjoying it in an atmosphere of excitement and fun will make it more meaningful in their research findings (48, 49). The role of the coach is very dominant here; coaches are perceived as life coaches for adolescents by adopting a more supportive approach, paying attention to the players’ needs, providing instructional feedback in a non-threatening manner, and praising the athletes; they seem to be able to reinforce the desire of adolescent athletes to develop (49). Coaches’ transformational leadership behavior and coach-athlete relationship quality are the best predictors of athlete development experiences (63).

In excitement, adolescent athletes also give meaning to new friends in the sports community by forming strong friendships that become a part of an adolescent's sports journey. Friends can provide feedback and motivate each other for improvement, and their skills create a drive to work harder and collaborate for team victories and success (49). Friendship and adult support in sports environments are woven over long periods, providing a sense of care that transcends organized relationships into enjoyable experiences for athletes’ well-being (48, 49). In its findings, sports become an opportunity to feel a sense of belonging and togetherness and blend into the crowd (51). Reflecting on the uniqueness of the sports experiences of adolescent athletes, the importance and strength of the friendships they create and maintain (48). Close friendships and the support and encouragement received from friends present in their sports experiences (52).

Thus, attention to field practice is significant to ensure that young athletes can develop through meaningful experiences participating in competitive sports. Practitioners and the adult environment of adolescent athletes must understand that the primary foundation for athletes starting in the world of sports is the enjoyment of adolescent athletes and meaningful friendships in competitive sports. Although they must be aware of the consequences of their participation, it becomes increasingly difficult to spend quality time with friends due to training commitments, and the nature of these relationships developing into potential sources of stress for adolescent athletes is a finding of the study (50, 53). Moreover, athletes learn from almost selective experiences of failure in competitive sports life, which significantly contributes to their growth; instead of giving up, they train harder to prove to their coaches that they are worthy (49). They see this pressure as an ‘opportunity’ for future challenges while understanding themselves as adolescent athletes. Of course, preventing and anticipating the negative consequences and meanings that can arise in the competitive sports lives of adolescent athletes and providing meaningful experiences in their activities to become elite athletes in the future will undoubtedly become a necessity.

### 4.2 Negative experiences as obstacles to athlete development of adolescent athletes

Prolong injuries in adolescent athletes are a negative experience for their long-term participation in competitive sports. Adolescent athletes experience an increase in training volume and intensity alongside the rising demands of training and competition, but this is also when they suffer the most injuries, leading to frustration and withdrawal from competitive sports (2, 55). Many athletes experience injury pain that develops gradually during training, such as from hamstring strains and ankle sprains (2, 54). In the findings (2), adolescent athletes recruited from the World Athletics Championships narrated that continuous training activities caused their injuries despite feeling pain to meet the competition and national team requirements (2). Adolescent athletes, consisting of national teams from six countries representing three continents (Africa, Europe, and Asia), also recounted that their parents wanted them to continue training despite injuries to avoid jeopardizing their future athletic careers. The athletes’ experiences also consistently mention broken or inadequate equipment, lack of warm-up, and improper technique during matches, which in turn lead to injuries. Supporting similar findings in many cases, poor facilities became the leading cause of their injuries and failure to reach high levels of competition (54, 55). Then, Poor handling, as concluded from a study that asked adolescent athletes to summarize their experiences with medical support, often leads young athletes to repeatedly state that they must manage their health issues (2, 64). The main points of the findings indicate that the risk of injury and the severity of injuries are significant concerns among elite adolescent athletes.

The understanding and caution of stakeholders, such as the social environment, play an important role in managing athlete injuries and athlete commitment in competitive sports. Social support from organizations that are transparent in fund allocation can help the commitment of young athletes (54, 64). However, the lack of professionalism within organizations (e.g., sports federations, sports managers/administrators) can hinder young athletes in developing and maintaining sports operations. The negative experiences of young athletes reporting insufficient funding from organizations to continue their sports, potentially burdening their parents or themselves, are the main reasons why most of them choose to leave sports early (38, 55).

The negative experiences of the elitist nature of high-performance sports impact their academic performance and disrupt their other life commitments, leading to conflicts of interest and attempts at other activities (38, 57). In some cases, negative experiences such as concerns about negative body image are common among the adolescent female athletes, ranging from mild dissatisfaction (e.g., body dissatisfaction) to more serious issues (e.g., eating disorders), and that the sports environment can either support or hinder the development of a positive body image (56). Not to mention the excessive pressure often exerted by parents to excel in their sports and win competitions, which sometimes leads to frustration instead of supporting the athletic performance of young athletes (38, 54). In line with previous literature, this study provides additional support showing that the social psychological climate affects the perception of commitment (65). High levels of social support and external regulation in the long term can influence their goals, enhance satisfaction, and help their commitment to sports activities (62). By explicitly highlighting the important role of social support in sports development practices, athlete well-being (e.g., physical, mental, social) is not independent but interconnected and must be deliberately pursued by social support stakeholders (66). The lack of relationships with adults and an unsupportive organizational environment for adolescent athletes create negative experiences that hinder them from continuing to commit to and participate in competitive sports in the long term.

### 4.3 The positive experience of competitive sports in the development of adolescent athletes

Changes in athlete development are often related to positive experiences in the sports activity environment. Coaches are responsible for creating a constructive training atmosphere where the coach-athlete relationship influences positive experiences that encompass trust, mutual understanding through discussion, and goal-oriented communication as key elements of positive experiences during childhood and adolescence (39, 40, 59). Coaches emphasize their desire to create an environment that facilitates player development through their experience and enthusiasm for the club and the sport (58). Such as validating findings that show the enjoyment of sports among adolescent athletes begins to fade due to excessive training with longer training distances and doing the same “work,” negative experiences with coaches or teammates (59). It is an art for a coach to continuously enhance an athlete's competence without diminishing their enjoyment and happiness in the atmosphere of competitive sports training.

When everything goes well, without interruptions, many young athletes focus their energy on athletic development and, to varying degrees, on their studies. In achieving those connections (parents, peers, coaches), they create stability for adolescent athletes in competitive sports (3). The family emerges as an introduction to sports for adolescent athletes and continues to motivate them to remain engaged in sports throughout childhood and adolescence (39, 58). The role of the family in several studies also ensures emotional support and self-esteem, attending training sessions, providing transportation to and from training, as well as tangible support and information through financial provision of equipment, supplies, and work-life balance (38, 39, 67–70). Equally important are relationships with friends who have similar experiences, which can foster a sense of belonging, need, understanding, and drive to continue improving (40, 58). In turn, connections with family, coaches, and friends significantly influence the maintenance of motivation and the consistent enjoyment of competitive sports, creating positive experiences in the development of young athletes.

Positive experiences in competitive sports have a transformative impact on the personal development of athletes. Scheduled activities such as eating, sleeping, and training take up a lot of time, so athletes rarely have the freedom to choose their activities, which becomes a valuable positive experience for future adolescent athletes (60). Adolescent athletes also explain how their self-confidence increases through participation in their respective sports teams, which influences the development of other skills, such as leadership, self-confidence, dedication, commitment, enjoyment, pride, and perseverance (3, 39, 60). Experience at the Youth Olympic Games (YOG) young athletes recounting the positive aspects of their participation such as: village environment, sports facilities, travel, ceremonies, medical services, and international experiences, sociocultural (especially informal) international, socio-cultural (especially informal) from the participation of young athletes in competitive sports (61). The participation of young athletes in competitive sports creates many positive experiences and, in turn, provides good personal development for young athletes for their future.

### 4.4 The social environment of adolescent athletes in competitive sports life

Understanding the significant experiences of adolescent athletes from the concept (Figure 3) may be beneficial in helping more adolescents engage in competitive sports while enhancing their positive developmental experiences. Confirming the theory that the characteristics of competitive sports training activities for adolescents reveal that consistent social support from coaches, parents, peers, and sports organization officials significantly contributes to relationships at the psychosocial development level during adolescence (50, 71, 72). At the same time, it supports the research model by Taryn K. Morgan, which describes how social support affects young people (73). For example, specific support system behaviors from coaches, parents, and peers influence the motivational atmosphere of adolescent athletes (74), creating a motivational climate that sustains athletes’ perseverance in sports (75, 76). In turn, it provides positive experiences by demonstrating trust and attachment to the sport and love for the sport they choose (77). Adolescent athletes speak very enthusiastically about how sports support autonomy and how meaningful and enjoyable they are for them (30, 78). The relationship with this social support fosters mutual understanding, trust, and friendship, culminating in the formation of harmonious connections that provide positive experiences in the sustainability of their competitive sports.

These findings align with the socio-ecological model (56, 79), where social support can address negative body image in adolescent girls’ sports. With a focus on negative experiences, improvements in all areas of the sports system may be valuable to help keep more adolescent female athletes engaged in sports while enhancing their positive sports experiences where misunderstandings in coaching adolescent athletes can transform the role of social support from coaches, parents, organizations, and peers into negative experiences in the competitive sports life of adolescents. As the theoretical findings from the Grounded Theory study of the influences affecting the sports experiences and withdrawal patterns of young athletes show, adolescents’ sports experiences and withdrawal patterns are influenced by their interpretations of personal, social, and organizational influences (80). Qualitative studies reveal that college, professional, and semi-professional athletes describe the theme of mental skill inhibition as consisting of athletes’ descriptions of poor coaches as distractions, causing self-doubt, demotivation, and team division (81). Similar to qualitative studies revealing that coaches are overly critical, negatively impacting their confidence during the transition period in elite sports (82). Athletes report that one of the main reasons they quit competitive sports is because they ‘do not like the pressure’ and ‘do not like their coach’ (83–85). Consistent studies on the withdrawal of adolescent athletes indicate that their negative experiences are influenced by their social environment, such as negative parental behaviors, which include placing too much emphasis on winning, having unrealistic expectations, and criticizing their children (76), deep sadness due to injuries, which can be caused by inadequate training programs (86, 87), poor coping techniques, and inadequate facilities (55, 88–90), conflicts with friends during training (84, 91).

Therefore, this becomes a concern in field practice to ensure that young athletes have positive experiences participating in competitive sports, which is undoubtedly greatly influenced by their social environment that works with their hearts for the development of young athletes—not just performing their duties and obligations without touching their hearts, for example, only focusing on teaching techniques, strategies, or physical aspects without considering the athletes’ emotions (63, 92–95). This is similar to parents who only accompany their child to the gym without asking, “How was your workout today?”. Give them space to grow and develop in competitive sports by experiencing positive experiences and reducing negative ones. If this process goes well, their transition to elite athletes will indeed be achieved and will benefit the personal development of young athletes.

### 4.5 Limitations of the review

A general consideration of this systematic review is the lack of literature comprehensively discussing adolescents experiencing organized competitive sports. The initial goal of this study is to synthesize various research projects that reveal important insights about adolescent athletes in their journey through organized competitive sports. The results of this synthesis are expected to enhance understanding and provide valuable insights into the significant experiences of adolescent athletes participating in competitive sports. This review is based on electronic databases, which apply secondary search strategies. This may have resulted in the identification and exclusion of several studies from the review. Finally, a total of 19 articles that met the inclusion criteria qualified, which is a modest evidence base, to examine the experiences of adolescent athletes. However, it is important to note several significant limitations. First of all, it is important to consider the heterogeneity of the participants, which can be seen from their ages. Secondly, competitive sports pursued by athletes have different characteristics, depending on whether individual- or team-oriented. Moreover, a deep understanding of the culture in which athletes are raised can make a significant difference, facilitating positive adaptation and development. The findings of this study will provide a broader understanding of adolescent athletes in competitive sports while also highlighting the importance of their socio-cultural development.

### 4.6 Implications for practice and future research

The findings suggest that working with young athletes requires attention to both visible and invisible factors. It is not just about tracking time, training loads, and medal targets. Young athletes need to be in the right environment, and the environment must be right for them to support positive development. Ensuring that sport provides meaning in their lives, fostering happiness, togetherness, and growth, is essential. They must connect with social elements such as family, coaches, and peers to support their journey towards becoming elite athletes. Future research should: (i) incorporate a wider range of methods to explore adolescent athletes in professional sports; (ii) investigate the long-term outcomes of both successful and unsuccessful adolescent athletes in professional sports; (iii) delve into the stories of adolescents being prepared to become elite athletes; and (iv) consider gender perspectives in competitive sport.

## 5 Conclusion

Three important characteristics of the experiences of adolescent athletes in competitive sports activities in their development through the available literature. First, it highlights how young athletes perceive their sport, experiencing deep happiness, friendships, and their challenges. Second, it emphasizes the negative experiences that become obstacles faced by young athletes in their development, such as injuries, weak commitment, and lack of social support, which adults often overlook. Third, it emphasizes the important positive experiences in life skills that athletes must develop through harmonious relationships with social elements that support their growth. From the significant experiences of adolescent athletes participating in competitive sports, the social environment contributes to each of those experiences. The practical implications of these findings indicate that working with young athletes is not only about training loads and medal goals but also about ensuring that they are in a supportive environment that fosters their development. To help adolescent athletes become elite, we must connect all the social elements within their sports environment. Future research should focus on developing a framework that highlights the social roles of young athletes and explores their personal experiences in a highly competitive sports environment, ultimately offering a comprehensive understanding of athlete development.

## Data Availability

The original contributions presented in the study are included in the article/Supplementary Material, further inquiries can be directed to the corresponding authors.

## References

[1] Merkel DL. Youth sport: positive and negative impact on young athletes. Open Access J Sports Med. (2013) 4:151–60. 10.2147/OAJSM.S33556 24379720 PMC3871410

[2] Timpka T Fagher K Bargoria V Gauffin H Andersson C Jacobsson J ‘The little engine that could’: a qualitative study of medical service access and effectiveness among adolescent athletics athletes competing at the highest international level. Int J Environ Res Public Health. (2021) 18(14):7278. 10.3390/ijerph18147278 34299729 PMC8304016

[3] Ronkainen N Aggerholm K Allen-Collinson J Ryba TV. Beyond life-skills: talented athletes, existential learning and (un)learning the life of an athlete. Qual Res Sport Exerc Health. (2023) 15(1):35–49. 10.1080/2159676X.2022.2037694

[4] Brower JJ. The professionalization of organized youth sport: social psychological impacts and outcomes. Ann Am Acad Pol Soc Sci. (1979) 445(1):39–46. 10.1177/000271627944500106

[5] Camiré M Santos F. Promoting positive youth development and life skills in youth sport: challenges and opportunities amidst increased professionalization. J Sport Pedagogy Res. (2019) 5(1):27–34.

[6] Sweeney L Horan D MacNamara Á. Premature professionalisation or early engagement? Examining practise in football player pathways. Front Sports Act Living. (2021) 3:660167. 10.3389/fspor.2021.660167 34164620 PMC8215134

[7] A. B. of Statistics. Children’s Participation in Cultural and Leisure Activities Australia. Canberra: Australian Bureau of Statistics (2000).

[8] Ryska T Hohensee D Cooley P Jones C. Participation motives in predicting sport dropout among Australian youth gymnasts. N Am J Psychol. (2002) 4(2):199–210.

[9] De Knop P Engstroem L-M Skirstad B Weiss MR. Worldwide Trends in Youth Sport. Champaign, IL: Human Kinetics (1996).

[10] Eime RM Harvey JT Sawyer NA Craike MJ Symons CM Polman RCJ Understanding the contexts of adolescent female participation in sport and physical activity. Res Q Exerc Sport. (2013) 84(2):157–66. 10.1080/02701367.2013.784846 23930541

[11] Eime RM Young JA Harvey JT Charity MJ Payne WR. A systematic review of the psychological and social benefits of participation in sport for children and adolescents: informing development of a conceptual model of health through sport. Int J Behav Nutr Phys Act. (2013) 10(1):1–21. 10.1186/1479-5868-10-98 23945179 PMC3751802

[12] Fraser-Thomas JL Côté J Deakin J. Youth sport programs: an avenue to foster positive youth development. Phys Educ Sport Pedagogy. (2005) 10(1):19–40. 10.1080/1740898042000334890

[13] Panza MJ Graupensperger S Agans JP Doré I Vella SA Evans MB. Adolescent sport participation and symptoms of anxiety and depression: a systematic review and meta-analysis. J Sport Exerc Psychol. (2020) 42(3):201–18. 10.1123/jsep.2019-0235 32438339 PMC7679280

[14] Patton GC Viner R. Pubertal transitions in health. Lancet. (2007) 369(9567):1130–9. 10.1016/S0140-6736(07)60366-3 17398312

[15] Lawrence D Johnson S Hafekost J Boterhoven de Haan K Sawyer M Ainley J The Mental Health of Children and Adolescents. Canberra: Department of Health (2015).

[16] Walton CC Rice S Hutter RIV Currie A Reardon CL Purcell R. Mental health in youth athletes: a clinical review. Adv Psychiatry Behav Health. (2021) 1(1):119–33. 10.1016/j.ypsc.2021.05.011

[17] Schubring A Thiel A. Growth problems in youth elite sports. Social conditions, athletes’ experiences and sustainability consequences. In: Barker-Ruchti N, Barker D, editors. Sustainability in High Performance Sport. London: Routledge (2017). p. 88–101. 10.4324/9781315657394

[18] Brettschneider W-D. Risks and opportunities: adolescents in top-level sport ñ growing up with the pressures of school and training. Eur Phy Educ Rev. (1999) 5(2):121–33. 10.1177/1356336X990052004

[19] Hayward FPI Knight CJ Mellalieu SD. A longitudinal examination of stressors, appraisals, and coping in youth swimming. Psychol Sport Exerc. (2017) 29:56–68. 10.1016/j.psychsport.2016.12.002

[20] Raedeke TD Smith AL. Coping resources and athlete burnout: an examination of stress mediated and moderation hypotheses. J Sport Exerc Psychol. (2004) 26(4):525–41. 10.1123/jsep.26.4.525

[21] Fry RW Morton AR Keast D. Overtraining in athletes: an update. Sports Med. (1991) 12:32–65. 10.2165/00007256-199112010-00004 1925188

[22] Grove JR Main LC Partridge K Bishop DJ Russell S Shepherdson A Training distress and performance readiness: laboratory and field validation of a brief self-report measure. Scand J Med Sci Sports. (2014) 24(6):e483–490. 10.1111/sms.12214 24646366

[23] Gilbert JN Gilbert W Morawski C. Coaching strategies for helping adolescent athletes cope with stress. J Phys Educ Recreat Dance. (2007) 78(2):13–24. 10.1080/07303084.2007.10597967

[24] Mountjoy M. International Olympic committee consensus statement: harassment and abuse (non-accidental violence) in sport. Br J Sports Med. (2016) 50(17):1019–29. 10.1136/bjsports-2016-096121 27118273

[25] Galli N Vealey RS. ‘Bouncing back’ from adversity: athletes’ experiences of resilience. Sport Psychol. (2008) 22(3):316–35. 10.1123/tsp.22.3.316

[26] Rutter M. Psychosocial resilience and protective mechanisms’. In: Rolf J Masten AS Cicchetti D Nuechterlein KH Weintraub S, editors. Risk and Protective Factors in the Development of Psychopathology. Cambridge: Cambridge Unversity Press (1990). p. 181–214.

[27] Bennie A O’Connor D. Athletic transition: an investigation of elite track and field participation in the post-high school years. Change Transform Ed. (2006) 9(1):59–68.

[28] Tamminen KA Holt NL. A meta-study of qualitative research examining stressor appraisals and coping among adolescents in sport. J Sports Sci. (2010) 28(14):1563–80. 10.1080/02640414.2010.512642 21058168

[29] Baker J Cote J Abernethy B. Sport-specific practice and the development of expert decision-making in team ball sports. J Appl Sport Psychol. (2003) 15(1):12–25. 10.1080/10413200305400

[30] Côté J Fraser-Thomas J. Youth involvement in sport. In: Crocker PRE, editor. Sport Psychology: A Canadian Perspective. Toronto: Pearson Prentice Hall (2007). p. 266–94.

[31] Besharat MA Pourbohlool S. Moderating effects of self-confidence and sport self-efficacyon the relationship between competitive anxietyand sport performance. Psychology. (2011) 2(07):760. 10.4236/psych.2011.27116

[32] Hays K Maynard I Thomas O Bawden M. Sources and types of confidence identified by world class sport performers. J Appl Sport Psychol. (2007) 19(4):434–56. 10.1080/10413200701599173

[33] Compton MT Koplan C Oleskey C Powers RA Pruitt D Wissow L. Prevention in Mental Health: an Introduction from the Prevention Committee of the Group for the Advancement of Psychiatry. Washington, DC: American Psychiatric Association Publishing (2010). p. 1–2. 10.1176/appi.books.9781615378029.lg01

[34] Lindner KJ Johns DP Butcher J. Factors in withdrawal from youth sport: a proposed model. J Sport Behav. (1991) 14(1):3. 10.1123/pes.1.3.195

[35] Weiss MR Petlichkoff LM. Children’s motivation for participation in and withdrawal from sport: identifying the missing links. Pediatr Exerc Sci. (1989) 1(3):195–211. 10.1123/pes.1.3.195 36949583

[36] Williams MD Braun LA Cooper LM Johnston J Weiss RV Qualy RL Hospitalized cancer patients with severe sepsis: analysis of incidence, mortality, and associated costs of care. Crit Care. (2004) 8(5):1–8. 10.1186/cc2893 15469571 PMC1065011

[37] Strachan L Côté J Deakin J. A new view: exploring positive youth development in elite sport contexts. Qual Res Sport Exerc Health. (2011) 3(1):9–32. 10.1080/19398441.2010.541483

[38] Thomas CE Chambers TP Main LC Gastin PB. Motives for dropout among former junior elite Caribbean track and field athletes: a qualitative investigation. Front Sports Act Living. (2021) 3:1–13. 10.3389/fspor.2021.696205 PMC8298833 34308348

[39] Thomas CE Chambers TP Main LC Gastin PB. Influencing the early development of world-class caribbean track and field athletes: a qualitative investigation Bursa, Turkiye. J Sports Sci Med. (2019) 18(4):758–71. 31827361 PMC6873121

[40] White RL Bennie A. Resilience in youth sport: a qualitative investigation of gymnastics coach and athlete perceptions. Int J Sports Sci Coach. (2015) 10(2–3):379–93. 10.1260/1747-9541.10.2-3.379

[41] Moher D Liberati A Tetzlaff J Altman DG, t PRISMA Group*. Preferred reporting items for systematic reviews and meta-analyses: the PRISMA statement. Ann Intern Med. (2009) 151(4):264–9. 10.7326/0003-4819-151-4-200908180-00135 19622511

[42] Gusenbauer M. Google scholar to overshadow them all? Comparing the sizes of 12 academic search engines and bibliographic databases. Scientometrics. (2019) 118(1):177–214. 10.1007/s11192-018-2958-5

[43] Page MJ McKenzie JE Bossuyt PM Boutron I Hoffmann TC Mulrow CD The PRISMA 2020 statement: an updated guideline for reporting systematic reviews. Br Med J. (2021) 372:n71. 10.1136/bmj.n71 33782057 PMC8005924

[44] Shaffril HAM Samah AA Samsuddin SF Ali Z. Mirror-mirror on the wall, what climate change adaptation strategies are practiced by the Asian’s fishermen of all? J Clean Prod. (2019) 232:104–17. 10.1016/j.jclepro.2019.05.262

[45] Donthu N Kumar S Mukherjee D Pandey N Lim WM. How to conduct a bibliometric analysis: an overview and guidelines. J Bus Res. (2021) 133:285–96. 10.1016/j.jbusres.2021.04.070

[46] Faber IR Bustin PMJ Oosterveld FGJ Elferink-Gemser MT Nijhuis-Van der Sanden MWG. Assessing personal talent determinants in young racquet sport players: a systematic review. J Sports Sci. (2016) 34(5):395–410. 10.1080/02640414.2015.1061201 26109450

[47] Letts L Wilkins S Law M Stewart D Bosch J Westmorland M. Guidelines for Critical Review Form: Qualitative Studies (Version 2.0). Hamilton: McMaster University Occupational Therapy Evidence-based Practice Research Group (2007). p. 1–12.

[48] McCalpin M Evans B Côté J. Young female soccer players’ perceptions of their modified sport environment. Sport Psychologist. (2017) 31(1):65–77. 10.1123/tsp.2015-0073

[49] Neely KC McHugh TLF Dunn JGH Holt NL. Athletes and parents coping with deselection in competitive youth sport: a communal coping perspective. Psychol Sport Exerc. (2017) 30:1–9. 10.1016/j.psychsport.2017.01.004

[50] Lisinskiene A Guetterman T Sukys S. Understanding adolescent–parent interpersonal relationships in youth sports: a mixed-methods study. Sports. (2018) 6(2):41. 10.3390/sports6020041 29910345 PMC6027542

[51] Mallinson-Howard SH Knight CJ Hill AP Hall HK. The 2×2 model of perfectionism and youth sport participation: a mixed-methods approach. Psychol Sport Exerc. (2018) 36:162–73. 10.1016/j.psychsport.2018.02.011

[52] Strachan L Davies K. Click! using photo elicitation to explore youth experiences and positive youth development in sport. Qual Res Sport Exerc Health. (2015) 7(2):170–91. 10.1080/2159676X.2013.867410

[53] Elliott S Drummond MJN Knight C. The experiences of being a talented youth athlete: lessons for parents. J Appl Sport Psychol. (2018) 30(4):437–55. 10.1080/10413200.2017.1382019

[54] Jacobsson J Bergin D Timpka T Nyce JM Dahlström Ö. Injuries in youth track and field are perceived to have multiple-level causes that call for ecological (holistic-developmental) interventions: a national sporting community perceptions and experiences. Scand J Med Sci Sports. (2018) 28(1):348–55. 10.1111/sms.12929 28605065

[55] Pérez BD Giménez AR Posadillo AÁS. Women and competitive sport: perceived barriers to equality. Cultura Ciencia Deporte. (2022) 17(54):63–86. 10.12800/ccd.v17i54.1887

[56] Koulanova A Sabiston CM Pila E Brunet J Sylvester B Sandmeyer-Graves A Ideas for action: exploring strategies to address body image concerns for adolescent girls involved in sport. Psychol Sport Exerc. (2021) 56:102017. 10.1016/j.psychsport.2021.102017

[57] Battaglia A Kerr G. Exploring sport stakeholders’ interpretations of the term dropout from youth sport. J Appl Sport Psychol. (2022) 34(1):67–88. 10.1080/10413200.2019.1707727

[58] Littlefair D Nichol D. Youth sport: a frontier in education. Front Educ (Lausanne). (2019) 4:1–6. 10.3389/feduc.2019.00119

[59] Larson HK McHugh TLF Young BW Rodgers WM. Pathways from youth to masters swimming: exploring long-term influences of youth swimming experiences. Psychol Sport Exerc. (2019) 41:12–20. 10.1016/j.psychsport.2018.11.007

[60] Bean C Kramers S Harlow M. Exploring life skills transfer processes in youth hockey and volleyball. Int J Sport Exerc Psychol. (2022) 20(1):263–82. 10.1080/1612197X.2020.1819369

[61] Parent MM Kristiansen E Macintosh EW. Athletes’ experiences at the youth Olympic games: perceptions, stressors, and discourse paradox. Event Management. (2014) 18(3):303–24. 10.3727/152599514X13989500765808

[62] Berki T Piko BF Page RM. The relationship between the models of sport commitment and self-determination among adolescent athletes. Acta Fac Ed Phys Univ Comen. (2019) 59(2):79–95. 10.2478/afepuc-2019-0007

[63] Vella SA Oades LG Crowe TP. The relationship between coach leadership, the coach–athlete relationship, team success, and the positive developmental experiences of adolescent soccer players. Phys Educ Sport Pedagogy. (2013) 18(5):549–61. 10.1080/17408989.2012.726976

[64] Fraser-Thomas J Côté J Deakin J. Understanding dropout and prolonged engagement in adolescent competitive sport. Psychol Sport Exerc. (2008) 9(5):645–62. 10.1016/j.psychsport.2007.08.003

[65] Hall MS Newland A Newton M Podlog L Baucom BR. Perceptions of the social psychological climate and sport commitment in adolescent athletes: a multilevel analysis. J Appl Sport Psychol. (Jan. 2017) 29(1):75–87. 10.1080/10413200.2016.1174906

[66] Berg BK Warner S. Advancing college athlete development via social support. J Issues Intercoll Athl. (2019) 12(1):87–113.

[67] Trussell DE Shaw SM. Organized youth sport and parenting in public and private spaces. Leis Sci. (2012) 34(5):377–94. 10.1080/01490400.2012.714699

[68] Scanlan TK Lewthwaite R. Social psychological aspects of competition for male youth sport participants: i. Predictors of competitive stress. J Sport Exerc Psychol. (1984) 6(2):208–26. 10.1123/jsp.6.2.208

[69] Dorsch TE Smith AL McDonough MH. Early socialization of parents through organized youth sport. Sport Exerc Perform Psychol. (2015) 4(1):3. 10.1037/spy0000021 19842542

[70] Howie EK Daniels BT Guagliano JM. Promoting physical activity through youth sports programs: it’s social. Am J Lifestyle Med. (2020) 14(1):78–88. 10.1177/1559827618754842 31903087 PMC6933572

[71] Sheridan D Coffee P Lavallee D. A systematic review of social support in youth sport. Int Rev Sport Exerc Psychol. (2014) 7(1):198–228. 10.1080/1750984X.2014.931999

[72] Wylleman P Lavallee D. A developmental perspective on transitions faced by athletes. Developmental Sport and Exercise Psychology: A Lifespan Perspective. Morgantown, WV: Fitness Information Technology (2004). p. 507–27.

[73] Morgan TK Giacobbi PR. Toward two grounded theories of the talent development and social support process of highly successful collegiate athletes. Sport Psychol. (2006) 20(3):295–313. 10.1123/tsp.20.3.295

[74] Keegan R Spray C Harwood C Lavallee D. The motivational atmosphere in youth sport: coach, parent, and peer influences on motivation in specializing sport participants. J Appl Sport Psychol. (2010) 22(1):87–105. 10.1080/10413200903421267

[75] Carr S. Adolescent–parent attachment characteristics and quality of youth sport friendship. Psychol Sport Exerc. (2009) 10(6):653–61. 10.1016/j.psychsport.2009.04.001

[76] Le Bars H Gernigon C Ninot G. Personal and contextual determinants of elite young athletes’ persistence or dropping out over time. Scand J Med Sci Sports. (2009) 19(2):274–85. 10.1111/j.1600-0838.2008.00786.x 18384491

[77] Baxter-Jones ADG Maffulli N. Parental influence on sport participation in elite young athletes. J Sports Med Phys Fitn. (2003) 43(2):250 5. 12853909

[78] Lafrenière M-AK Jowett S Vallerand RJ Carbonneau N. Passion for coaching and the quality of the coach–athlete relationship: the mediating role of coaching behaviors. Psychol Sport Exerc. (2011) 12(2):144–52. 10.1016/j.psychsport.2010.08.002

[79] Bronfenbrenner U Morris PA. The ecology of developmental processes. In: Damon W, Lerner RM, editors. Handbook of Child Psychology: Theoretical Models of Human Development. Vol. 1. New York, NY: John Wiley & Sons, Inc. (1998). p. 993–1028.

[80] Battaglia A Kerr G Tamminen K. A grounded theory of the influences affecting youth sport experiences and withdrawal patterns. J Appl Sport Psychol. (2022) 34(4):780–802. 10.1080/10413200.2021.1872732

[81] Gearity BT Murray MA. Athletes’ experiences of the psychological effects of poor coaching. Psychol Sport Exerc. (2011) 12(3):213–21. 10.1016/j.psychsport.2010.11.004

[82] Bruner MW Munroe-Chandler KJ Spink KS. Entry into elite sport: a preliminary investigation into the transition experiences of rookie athletes. J Appl Sport Psychol. (2008) 20(2):236–52. 10.1080/10413200701867745

[83] Salguero A Gonzalez-Boto R Tuero C Marquez S. Identification of dropout reasons in young competitive swimmers. J Sports Med Phys Fitn. (2003) 43(4):530–4. 14767416

[84] Evans L Hardy L. Sport injury and grief responses: a review. J Sport Exerc Psychol. (1995) 17(3):227–45. 10.1123/jsep.17.3.227

[85] Ruggiero TE Lattin KS. Intercollegiate female coaches’ use of verbally aggressive communication toward African American female athletes. Howard J Commun. (2008) 19(2):105–24. 10.1080/10646170801990946

[86] Protivnak JJ Tessmer SS Lockard NL Spisak T. Unrecognized grief: counseling interventions for injured student-athletes. J Counselor Practice. (2023) 14(1):75–101. 10.22229/aws8975026

[87] Van der Poel J Nel P. Relevance of the kübler-ross model to the post-injury responses of competitive athletes. S Afr J Res Sport Phys Ed Recreation. (2011) 33(1):151–64. 10.4314/sajrs.v33i1.65496

[88] Diekfuss JA. Can we capitalize on central nervous system plasticity in young athletes to inoculate against injury? J Sci Sport Exerc. (2020) 2(4):305–18. 10.1007/s42978-020-00080-3

[89] Jowett S. Coaching effectiveness: the coach–athlete relationship at its heart. Curr Opin Psychol. (2017) 16:154–8. 10.1016/j.copsyc.2017.05.006 28813341

[90] Von Rosen P Heijne A Frohm A Fridén C Kottorp A. High injury burden in elite adolescent athletes: a 52-week prospective study. J Athl Train. (2018) 53(3):262–70. 10.4085/1062-6050-251-16 29412695 PMC5894377

[91] Mutz M. Athletic participation and the approval and use of violence: a comparison of adolescent males in different sports disciplines. Eur J Sport Soc. (2012) 9(3):177–201. 10.1080/16138171.2012.11687896

[92] Jowett S Chaundy V. An investigation into the impact of coach leadership and coach-athlete relationship on group cohesion. Group Dyn Theory Res Pract. (2004) 8(4):302. 10.1037/1089-2699.8.4.302

[93] Jowett S Poczwardowski A. Understanding the coach-athlete relationship. In: Jowette S, Lavallee D, editors. Social Psychology in Sport. Champaign, IL: Human Kinetics (2007). p. 3–14. 10.5040/9781492595878.ch-001

[94] Nash C Collins D. Tacit knowledge in expert coaching: science or art? Quest. (2006) 58(4):465–77. 10.1080/00336297.2006.10491894

[95] Nash CS Sproule J Horton P. Excellence in coaching: the art and skill of elite practitioners. Res Q Exerc Sport. (2011) 82(2):229–38. 10.5641/027013611X13119541883744 21699102

